# African traditional medicine: historical legacy, colonial disruption, and pathways for integration into modern healthcare

**DOI:** 10.3389/fphar.2026.1842857

**Published:** 2026-06-01

**Authors:** Ermias Mergia Terefe

**Affiliations:** Department of Pharmacology, Pharmacognosy and Pharmaceutical Chemistry, School of Pharmacy and Health Sciences, United States International University-Africa, Nairobi, Kenya

**Keywords:** african medicines agency, african traditional medicine, ethnopharmacology, health systems, integration, medicinal plants, pharmacological mechanisms, traditional medicine policy

## Abstract

African Traditional Medicine (ATM) remains an important component of healthcare across Africa and continues to play a significant role within pluralistic healthcare systems where traditional and biomedical practices coexist. This narrative review critically examines the historical development, ethnopharmacological relevance, scientific validation, integration models, governance frameworks, and ethical considerations surrounding ATM within contemporary African healthcare systems. A structured literature search was conducted using PubMed, Scopus, Web of Science, Google Scholar, and institutional policy sources including publications from the World Health Organization (WHO), African Union (AU), and African Medicines Agency (AMA). The literature was synthesized thematically to examine historical foundations, indigenous knowledge systems, medicinal resources, clinical evidence, healthcare integration, regulation, and policy development related to ATM. The review demonstrates that ATM encompasses diverse therapeutic modalities including herbal medicine, spiritual and ritual healing, manual therapies, traditional birth attendance, and community-based preventive care. Africa’s extensive medicinal biodiversity and indigenous knowledge systems provide substantial ethnopharmacological potential for natural-product research and drug discovery. However, integration of ATM into formal healthcare systems remains constrained by limited clinical validation, challenges in standardization and quality control, fragmented regulatory systems, insufficient research infrastructure, and persistent epistemological tensions between biomedical and indigenous healthcare paradigms. The review further highlights the influence of colonial disruption on the marginalization of indigenous medical knowledge and examines evolving efforts toward integration through institutional, collaborative, referral-based, and regulated product models. Regional and international governance initiatives, including WHO strategies and the African Medicines Agency, are increasingly promoting harmonized regulation, practitioner oversight, and evidence-informed integration of traditional medicine across Africa. Ultimately, effective integration of ATM requires interdisciplinary research, strengthened regulatory frameworks, ethical governance, biodiversity conservation, and equitable protection of indigenous knowledge systems. Balanced and context-specific approaches that combine scientific rigor with cultural responsiveness may contribute to safer, more inclusive, and sustainable healthcare systems within contemporary African contexts.

## Introduction

African Traditional Medicine (ATM) refers to diverse indigenous healthcare knowledge systems, therapeutic practices, and healing traditions developed by African communities over centuries for the prevention, diagnosis, and management of physical, mental, social, and spiritual illnesses. The World Health Organization defines traditional medicine as the sum of knowledge, skills, and practices based on indigenous theories, beliefs, and experiences used in maintaining health and treating disease (Atanasov et al., 2021; WHO, 2025). In the African context, ATM is not a single uniform medical system but rather a heterogeneous and context-specific network of healing traditions shaped by cultural beliefs, spirituality, ecological diversity, and community-based understandings of health and illness (Akpuogwu et al., 2025; WHO, 2025). These systems are dynamic and evolving rather than static cultural traditions, and their practices, effectiveness, and institutional recognition vary considerably across regions and communities.

ATM is grounded in indigenous knowledge systems, which refer to locally developed systems of knowledge generation, transmission, and application that evolve through long-term interaction between communities and their social and environmental contexts. Such knowledge is commonly transmitted through oral traditions, apprenticeship, observation, and experiential learning across generations. In healthcare, indigenous knowledge systems encompass not only therapeutic interventions but also culturally embedded understandings of disease causation, healing, prevention, and the relationship between humans, nature, and spirituality (Lamwaka et al., 2024).

Importantly, ATM comprises several distinct but interconnected therapeutic modalities. Herbal medicine, the most extensively studied component, involves the use of medicinal plants and plant-derived preparations for therapeutic purposes. Spiritual and ritual healing addresses illness through practices such as divination, prayers, rituals, and ancestral consultation, particularly where illness is understood to have psychosocial or spiritual dimensions. Manual therapies include bone setting, massage, scarification, and physical manipulation, while traditional birth attendance encompasses maternal and neonatal care provided within community settings. ATM also includes preventive and community-based practices related to hygiene, nutrition, behavioral regulation, and environmental protection that contribute to collective health and social wellbeing (D’Almeida et al., 2024; Ikhoyameh et al., 2024; Terefe et al., 2022). Although analytically distinguishable, these modalities frequently operate in an integrated manner within many African healthcare traditions.

The concept of integration in this review refers to the incorporation of traditional medicine into formal healthcare systems through clinical, regulatory, educational, and policy frameworks. Integration may range from parallel coexistence between traditional and biomedical systems to collaborative referral structures, practitioner licensing, institutional recognition, and inclusion within national health strategies. Importantly, integration does not necessarily imply assimilation of ATM into biomedicine, but rather the development of pluralistic healthcare systems capable of accommodating multiple therapeutic paradigms while ensuring patient safety and evidence-informed practice (Wang et al., 2025; WHO, 2025).

Modern healthcare, as used in this review, refers primarily to contemporary biomedical systems characterized by standardized diagnostics, evidence-based clinical practice, pharmacological therapies, institutionalized professional training, and formal regulatory oversight. While biomedical healthcare has substantially improved disease management and public health outcomes, its historical dominance within African healthcare systems has often marginalized indigenous therapeutic systems and alternative epistemologies of healing (Ahlberg, 2017; Degu A et al., 2022).

The contemporary position of ATM cannot be fully understood without reference to colonial disruption. Colonial administrations institutionalized Western biomedicine through legal, educational, religious, and political systems while frequently suppressing or delegitimizing indigenous African healing practices. Missionary medicine and colonial medical education often portrayed traditional healing as unscientific or superstitious, while legal instruments such as Witchcraft Acts criminalized aspects of indigenous therapeutic practice (Abdullahi, 2011; Feierman and Janzen, 1998; Mthethwa et al., 2025; Noor and Terefe, 2025). These historical processes contributed to enduring epistemological hierarchies in which biomedical knowledge became institutionally dominant while indigenous systems remained marginalized within research, regulation, and healthcare governance.

Despite this historical marginalization, ATM remains widely utilized across Africa, particularly in settings where biomedical services are geographically inaccessible, financially unaffordable, or culturally incongruent. However, utilization patterns vary substantially across countries, populations, and disease conditions, and many individuals engage in medical pluralism by simultaneously or sequentially using both traditional and biomedical healthcare systems (Berhe et al., 2024).

Africa’s rich biodiversity further contributes to the significance of ATM. The continent contains thousands of medicinal plant species with important ethnopharmacological and therapeutic potential. Nevertheless, much of this knowledge remains incompletely documented and insufficiently translated into clinically validated and standardized therapeutic products (Atanasov et al., 2021; WHO, 2025).

Against this background, a contemporary review of ATM must move beyond generalized narratives of cultural relevance and accessibility to critically examine its historical foundations, epistemological positioning, ethnopharmacological evidence, scientific validation, integration pathways, and governance challenges within modern healthcare systems. This review therefore explores the historical evolution of ATM, its scientific and ethnopharmacological foundations, models of healthcare integration, regulatory and ethical considerations, and the opportunities and barriers associated with developing safe, equitable, and evidence-informed approaches to ATM within contemporary African health systems (Atanasov et al., 2021; D’Almeida et al., 2024; Wang et al., 2025; WHO, 2025).

## Methods

This study was conducted as a narrative review examining the historical development, ethnopharmacological relevance, scientific evidence, integration pathways, governance frameworks, and ethical considerations of African Traditional Medicine (ATM) within contemporary healthcare systems. A narrative review approach was considered appropriate because the subject spans multiple interdisciplinary domains, including ethnopharmacology, medical anthropology, history, public health, health policy, healthcare systems research, and indigenous knowledge studies.

A targeted literature search was conducted between January and March 2026 using electronic databases including PubMed, Scopus, Web of Science, and Google Scholar. Additional policy and institutional documents were identified through targeted searches of reports and publications from organizations such as the World Health Organization (WHO), African Union (AU), African Medicines Agency (AMA), and regional health bodies.

Search terms included combinations of “African traditional medicine,” “traditional medicine Africa,” “ethnopharmacology,” “medicinal plants Africa,” “integration,” “indigenous knowledge systems,” “colonial medicine,” “traditional medicine regulation,” “drug discovery,” “clinical trials,” and “African Medicines Agency.” Boolean operators (AND/OR) were used to refine searches across databases.

Priority was given to literature published between 2022 and 2025 in order to capture recent developments in ethnopharmacology, healthcare integration, regulation, and policy related to ATM. Earlier foundational publications were also included where necessary to provide historical, conceptual, and theoretical context.

Eligible sources included peer-reviewed original research articles, systematic and scoping reviews, ethnopharmacological studies, policy documents, regulatory reports, and authoritative institutional publications relevant to ATM. The literature search yielded a broad range of potentially relevant sources from electronic databases and institutional publications. Following removal of duplicates and screening for relevance and alignment with the objectives of the review, sources considered most relevant to the historical, ethnopharmacological, regulatory, and healthcare integration dimensions of ATM were included in the final narrative synthesis. Screening and thematic synthesis were conducted by the author.

The retrieved literature was synthesized thematically across major domains including historical development, indigenous knowledge systems, colonial disruption, ethnopharmacology, scientific validation, healthcare integration, governance frameworks, and ethical considerations. Particular attention was given to recurring themes, emerging policy developments, interdisciplinary perspectives, and evolving approaches to integration of ATM into contemporary healthcare systems.

As this was a narrative review, the protocol was not registered, and formal risk-of-bias assessment was not performed. The review is therefore subject to limitations associated with narrative synthesis, including potential selection bias, heterogeneity of included evidence sources, and variability in methodological quality across studies. Nevertheless, the narrative review design was considered appropriate for providing a broad conceptual, historical, scientific, and policy-oriented synthesis of African Traditional Medicine.

## Historical legacy of African traditional medicine

African Traditional Medicine (ATM) represents one of the oldest continuously practiced healthcare traditions globally and is deeply embedded within the cultural, social, spiritual, and historical development of African societies. Long before the institutionalization of Western biomedicine, African communities had established organized systems of healthcare grounded in indigenous knowledge, empirical observation, environmental adaptation, and culturally mediated understandings of illness and healing. These systems evolved dynamically across different ecological and sociocultural contexts and should not be interpreted as static or homogeneous traditions, but rather as adaptive and context-specific healthcare systems shaped by local histories and community experiences (Mutombo et al., 2023; Ouma, 2022).

### Indigenous knowledge systems and healthcare traditions

The historical foundation of ATM lies in indigenous knowledge systems transmitted primarily through oral traditions, apprenticeship, observation, ritual practice, and intergenerational learning. Knowledge transmission was often structured through prolonged mentorship under experienced healers, herbalists, diviners, spiritual leaders, and traditional birth attendants. Apprenticeship systems commonly involved practical training in medicinal plant identification, preparation methods, diagnostic approaches, spiritual practices, and ethical responsibilities within community healthcare structures (Yifru et al., 2025).

In many African societies, medicinal knowledge was preserved within family lineages, clan systems, or specialized healer networks, where access to particular therapeutic knowledge could be restricted to initiated members. These structures functioned not only as mechanisms of knowledge preservation but also as systems of professional legitimacy, social accountability, and quality control. Traditional healers occupied influential social roles that extended beyond therapeutic practice to include counseling, spiritual mediation, conflict resolution, and community guidance (Hooft et al., 2020).

ATM historically incorporated diverse but interconnected therapeutic modalities, including herbal medicine, spiritual and ritual healing, manual therapies, traditional birth attendance, and preventive community-based healthcare practices. These approaches frequently operated within holistic frameworks that viewed health as a dynamic balance between biological, social, environmental, and spiritual factors. Illness was therefore often interpreted not solely as a biological disturbance but also as a disruption of communal, moral, or spiritual equilibrium (Lamwaka et al., 2024).

This holistic orientation shares certain similarities with contemporary biopsychosocial approaches to healthcare in recognizing the interaction between physical, psychological, social, and environmental determinants of health. However, ATM differs from biomedical systems through its explicit incorporation of spirituality, ancestral relationships, and communal identity into concepts of illness and healing. Consequently, evaluating ATM exclusively through reductionist biomedical paradigms may inadequately capture its therapeutic logic, cultural significance, and experiential dimensions (Wang et al., 2025; WHO, 2025).

### Colonial disruption and epistemological transformation

The historical trajectory of ATM was profoundly altered by colonial expansion and the institutionalization of Western biomedicine across Africa. Colonial administrations introduced biomedical hospitals, missionary medicine, and formal medical education systems modeled on European healthcare structures. Indigenous healing practices were frequently portrayed as primitive, irrational, or superstitious, while Western biomedicine was promoted as the legitimate and scientifically superior form of healthcare (Abdullahi, 2011; Feierman and Janzen, 1998; Mthethwa et al., 2025).

Missionary medicine played a central role in reshaping colonial healthcare systems by discouraging or condemning many traditional healing practices, particularly spiritual and ritual dimensions associated with indigenous belief systems. Colonial educational institutions further reinforced Western scientific paradigms while excluding indigenous medical knowledge from formal curricula, research, and institutional recognition.

In several colonial territories, legal frameworks such as Witchcraft Acts criminalized aspects of traditional healing and ritual practice, contributing to the stigmatization and marginalization of indigenous practitioners. These processes disrupted pre-existing systems of knowledge transmission, weakened healer networks, and reduced the institutional legitimacy of ATM within emerging colonial healthcare structures (Kabumba, 2023).

Importantly, colonial disruption extended beyond healthcare delivery to broader epistemological transformation. Western biomedical knowledge became institutionally privileged within education, governance, regulation, and scientific research, while indigenous knowledge systems were marginalized within formal policy and academic institutions. These historical processes created enduring knowledge hierarchies that continue to influence research priorities, healthcare governance, professional legitimacy, and integration of ATM into contemporary health systems (Ikhoyameh et al., 2024).

### Regional diversity and contextual variation

ATM remains highly heterogeneous across the African continent, reflecting ecological diversity, cultural variation, linguistic differences, and historical influences. Therapeutic systems in West Africa often emphasize herbal medicine combined with spiritual healing practices, whereas East African traditions may integrate herbal medicine with faith-based approaches. In Southern Africa, divination and ancestor-mediated healing frequently play central roles, while North African medical traditions demonstrate historical influence from Greco-Arabic (Unani) medicine (D’Almeida et al., 2024; Devine et al., 2022; Krah et al., 2018).

These regional variations have important implications for ethnopharmacological research, healthcare integration, and policy development. Differences in medicinal plant use, therapeutic philosophies, preparation methods, and cultural interpretations of illness mean that generalized representations of ATM are often insufficient. Context-specific approaches are therefore necessary for meaningful scientific evaluation, regulatory development, and integration of ATM into contemporary African healthcare systems (Ampomah et al., 2020; Wang et al., 2025; WHO, 2025).

## Ethnopharmacology and medicinal resources

Ethnopharmacology examines the medicinal use of natural substances within traditional knowledge systems and provides an important interface between indigenous therapeutic practices and modern biomedical research. In the African context, ethnopharmacology links extensive biodiversity with longstanding ethnomedical knowledge developed through centuries of empirical observation, cultural transmission, and community-based healthcare practice. African Traditional Medicine (ATM) therefore represents both a healthcare resource and a significant source of pharmacologically active natural products with potential relevance for contemporary drug discovery and therapeutic development (Atanasov et al., 2021).

### African biodiversity and medicinal resources

Africa is among the world’s most biologically diverse regions, containing an estimated 45,000–60,000 vascular plant species, of which approximately 5,000–7,000 are used medicinally within traditional healthcare systems (Nargawe et al., 2023). These medicinal resources are used in the management of infectious diseases, inflammatory conditions, metabolic disorders, reproductive health problems, mental illnesses, and chronic non-communicable diseases (Etaware et al., 2025).

The therapeutic significance of African medicinal plants extends beyond traditional healthcare use. Medicinal plant resources also represent important reservoirs for natural-product chemistry, ethnopharmacological research, and pharmaceutical innovation. However, only a relatively small proportion of African medicinal plants has undergone comprehensive phytochemical characterization, pharmacological evaluation, toxicological assessment, or clinical investigation. Much of the associated indigenous knowledge remains locally held, orally transmitted, and incompletely documented, limiting its accessibility for systematic scientific study and translational research (Lamwaka et al., 2024; Sifuna and Sifuna, 2022).

Environmental pressures further threaten the sustainability of medicinal resources across the continent. Deforestation, overharvesting, habitat degradation, climate change, and biodiversity loss increasingly affect the availability of medicinal plant species and contribute to erosion of indigenous therapeutic knowledge systems. These challenges have important implications for conservation, healthcare sustainability, and future ethnopharmacological research (Ikhoyameh et al., 2024).

### Medicinal plants and bioactive compounds

African medicinal plants contain diverse classes of bioactive compounds including alkaloids, flavonoids, terpenoids, polyphenols, tannins, glycosides, and saponins. These compounds exhibit a broad range of pharmacological activities such as antimicrobial, anti-inflammatory, antioxidant, antidiabetic, anticancer, antiparasitic, and immunomodulatory effects. Unlike many conventional pharmaceuticals based on isolated single molecules, traditional herbal preparations frequently contain multiple phytochemicals capable of interacting synergistically across several biological pathways (Saeed et al., 2025).

Several African medicinal plants illustrate the relationship between traditional therapeutic use and pharmacological activity. *Cryptolepis sanguinolenta*, widely used in West African traditional medicine for febrile illnesses and malaria, contains cryptolepine, an indoloquinoline alkaloid with antiplasmodial, antimicrobial, and anti-inflammatory properties. *Prunus africana* contains phytosterols and pentacyclic triterpenes that have been investigated in the management of benign prostatic hyperplasia due to their anti-inflammatory and anti-proliferative effects (Karim et al., 2025; Komakech et al., 2017; Terefe et al., 2022).

Other medicinal plants demonstrate relevance in chronic inflammatory and metabolic disorders. *Vernonia amygdalina* contains flavonoids and sesquiterpene lactones associated with antioxidant, antidiabetic, and anticancer activity, while *Moringa oleifera* contains isothiocyanates and flavonoids with anti-inflammatory and cardioprotective effects (Degu S et al., 2024).

Additional examples further illustrate the pharmacological diversity of African medicinal resources. *Zanthoxylum chalybeum* possesses alkaloids and limonoids with antiplasmodial and antibacterial activity, whereas *Azadirachta indica* (Neem) demonstrates antimalarial, antiviral, and immunomodulatory effects associated with limonoids such as azadirachtin (Mguni et al., 2023; Nagini et al., 2024). *Securidaca longipedunculata* contains methyl salicylate and xanthones with analgesic and antimicrobial activity, while *Warburgia salutaris* produces drimane sesquiterpenes with antimicrobial and antifungal properties (Meddows-Taylor and Ramadwa, 2025).

Collectively, these examples demonstrate that African medicinal plants are not only culturally significant but also biologically active, with mechanisms that frequently align with recognized biomedical targets. Traditional therapeutic knowledge has therefore contributed substantially to identifying plants with potential pharmacological relevance and may improve the efficiency of natural-product research and drug discovery efforts.

### Pharmacological complexity and translational relevance

An important characteristic of many traditional herbal formulations is their phytochemical complexity. Traditional remedies often contain multiple bioactive constituents capable of interacting synergistically rather than acting through a single isolated mechanism. This multidimensional pharmacological activity may partly explain the continued therapeutic use of certain herbal preparations in chronic and multifactorial conditions.

At the same time, phytochemical complexity presents scientific challenges related to reproducibility, standardization, dose optimization, and quality control. Variability in plant species, environmental conditions, harvesting practices, preparation methods, and storage conditions can significantly influence phytochemical composition and therapeutic activity. These factors complicate clinical validation and development of standardized phytopharmaceutical products.

Despite these limitations, African ethnopharmacology remains an important area of scientific and therapeutic relevance. The continent’s medicinal resources continue to provide opportunities for natural-product research, drug discovery, and development of evidence-informed traditional medicines, while also highlighting the importance of biodiversity conservation and preservation of indigenous medical knowledge systems (Atanasov et al., 2021; Wang et al., 2025).

## Scientific validation and standardization

Scientific validation is central to evaluating the safety, efficacy, quality, and reproducibility of African Traditional Medicine (ATM). While many traditional therapies have longstanding histories of community use, broader scientific recognition increasingly depends on systematic pharmacological, toxicological, clinical, and quality-control investigations (WHO, 2025).

Current evidence on African medicinal plants derives primarily from ethnobotanical documentation, phytochemical studies, *in vitro* experiments, and animal models. These studies have identified numerous bioactive compounds with antimicrobial, anti-inflammatory, antioxidant, antiparasitic, anticancer, and immunomodulatory properties. However, relatively few African herbal medicines have undergone rigorous human clinical trials or advanced through standardized drug-development pathways (Agu et al., 2025; Atanasov et al., 2021).

One of the major scientific challenges involves the complexity and variability of traditional herbal formulations. Unlike many conventional pharmaceuticals based on isolated active compounds, ATM preparations frequently contain multiple phytochemical constituents whose composition may vary according to plant species, geographical origin, harvesting conditions, preparation methods, storage practices, and dosage forms. Such variability can significantly influence therapeutic activity, reproducibility, and safety profiles across studies (Ouma, 2022).

Standardization is therefore essential for improving scientific reliability and reproducibility. This includes authentication of medicinal plant species, phytochemical profiling, identification of active constituents, contaminant screening, microbial testing, stability assessment, and dose standardization. Frameworks such as Good Agricultural and Collection Practices (GACP) and Good Manufacturing Practices (GMP) are increasingly important for improving consistency and quality assurance of herbal medicines (WHO, 2025).

Toxicological evaluation is equally important because natural origin does not necessarily guarantee safety. Certain medicinal plants may produce adverse effects related to intrinsic toxicity, contamination, inappropriate dosing, prolonged use, or herb–drug interactions. Consequently, systematic toxicological assessment and safety monitoring remain essential components of scientific evaluation of African herbal medicines (Ikhoyameh et al., 2024).

Clinical evaluation of ATM also presents methodological challenges. Conventional randomized controlled trials may not fully capture the complex and individualized nature of some traditional therapies, particularly where treatment involves combinations of herbal, spiritual, social, and behavioral interventions. As a result, complementary approaches such as observational studies, pragmatic clinical trials, and community-based research may contribute valuable evidence regarding therapeutic effectiveness and patient experience (Lamwaka et al., 2024).

## Integration models and healthcare system frameworks

The integration of African Traditional Medicine (ATM) into contemporary healthcare systems has become an increasingly important topic within public health, health policy, and healthcare governance discussions across Africa. Integration refers to the structured incorporation of traditional medicine into formal healthcare systems through regulatory, clinical, educational, and institutional mechanisms. However, integration is not a single uniform process; rather, it encompasses multiple models of interaction between traditional and biomedical healthcare systems that vary according to historical context, policy priorities, regulatory capacity, and sociocultural factors (WHO, 2025).

### Parallel healthcare model

In many African countries, ATM and biomedical healthcare systems continue to operate largely in parallel with limited formal interaction. Under this model, patients independently move between traditional and biomedical providers based on accessibility, affordability, cultural beliefs, perceived effectiveness, and illness type. Traditional healers and biomedical practitioners function separately, and formal referral systems or institutional collaboration are minimal. Although this model allows coexistence of multiple therapeutic systems, lack of coordination may contribute to fragmented care, delayed communication, and increased risk of unmonitored concurrent use of herbal and conventional medicines (Ampomah et al., 2020; Krah et al., 2018; Wang et al., 2025).

### Referral-based integration

Referral-based integration involves structured or informal referral relationships between traditional healers and biomedical practitioners. In this framework, traditional practitioners may refer patients requiring biomedical intervention, while biomedical providers may acknowledge or accommodate patients’ use of traditional therapies. Referral systems have been explored particularly in areas such as maternal health, mental health, HIV/AIDS care, chronic disease management, and community-based healthcare delivery. Such approaches aim to improve continuity of care while recognizing the continued social legitimacy and accessibility of traditional healers within many communities (Hooft et al., 2020).

### Collaborative healthcare model

The collaborative model involves more active cooperation between traditional and biomedical practitioners through communication, interdisciplinary engagement, and shared patient management. Under this approach, healthcare providers maintain distinct professional roles but participate in coordinated care pathways designed to improve patient safety and therapeutic outcomes. Collaborative models may include joint training programs, interdisciplinary consultations, shared health education initiatives, and integration of culturally informed care practices within formal healthcare systems (Ahlberg, 2017; Wang et al., 2025). This model is increasingly discussed within pluralistic healthcare systems because many patients simultaneously utilize both traditional and biomedical therapies. Improved communication between practitioners may therefore help reduce risks associated with herb–drug interactions, delayed diagnosis, and fragmented treatment pathways while promoting culturally responsive healthcare delivery (Ikhoyameh et al., 2024).

### Institutional integration model

Institutional integration represents a more formalized approach in which traditional medicine services are incorporated directly into healthcare institutions such as hospitals, clinics, universities, or primary healthcare systems. This may include establishment of traditional medicine units within hospitals, practitioner licensing systems, formal educational programs, research centers, and inclusion of traditional medicine services within national health strategies (Ahlberg, 2017; Vasconi and Owoahene-Acheampong, 2011; WHO, 2025). Several African countries have introduced elements of institutional integration. Ghana has established traditional medicine units within selected hospitals and developed institutional structures such as the Centre for Plant Medicine Research to support herbal medicine research and product development. Nigeria has similarly advanced institutional support through the Nigerian Natural Medicine Development Agency (NNMDA), which promotes research, standardization, and policy development related to traditional medicine (Oluwafemi, 2025; Vasconi and Owoahene-Acheampong, 2011). In East Africa, integration efforts remain more limited but continue to evolve. Ethiopia formally recognizes traditional medicine within national health policy frameworks, and academic institutions increasingly engage in medicinal plant and ethnopharmacological research. Kenya has also introduced policy and regulatory initiatives related to herbal and complementary medicines, while universities and research institutions contribute to documentation and scientific evaluation of medicinal plants (Ahlberg, 2017; Balcha, 2025; Gakuya et al., 2020).

### Regulated product integration

Another important integration framework focuses on regulation and commercialization of herbal medicines and traditional medical products. Under this model, traditional medicinal formulations undergo processes of quality control, standardization, safety assessment, and regulatory approval before entering formal pharmaceutical markets. This approach emphasizes product regulation rather than direct institutional integration of traditional healing practices themselves. Regulated product models increasingly align with broader continental and global policy initiatives aimed at strengthening safety monitoring, pharmacovigilance, manufacturing standards, and harmonized regulatory oversight of herbal medicines. However, implementation remains uneven across many African countries due to differences in institutional capacity, legislation, research infrastructure, and regulatory enforcement (Ampomah et al., 2020; Mutombo et al., 2023; Wang et al., 2025; WHO, 2025).

### Challenges and considerations in integration

Despite increasing policy interest in integration, coordination between traditional and biomedical healthcare systems remains complex due to differences in therapeutic philosophies, training systems, and professional structures across healthcare settings. Effective integration therefore requires context-specific approaches that balance patient safety and scientific accountability with cultural respect and recognition of indigenous healthcare systems (Wang et al., 2025; WHO, 2025).

## Policy, governance, and regulatory frameworks

Policy and regulatory frameworks play a central role in determining how African Traditional Medicine (ATM) is recognized, governed, and integrated within contemporary healthcare systems. Across Africa, regulatory approaches remain highly heterogeneous, reflecting differences in national legislation, institutional capacity, political commitment, and health system priorities. While some countries have developed formal structures for traditional medicine governance, others continue to rely on fragmented or weakly enforced frameworks (WHO, 2025). At the global level, the World Health Organization (WHO) has provided strategic guidance for the development and regulation of traditional medicine. The WHO Global Traditional Medicine Strategy 2025–2034 emphasizes the need to strengthen governance, regulatory oversight, practitioner accountability, safety monitoring, and integration of traditional medicine within universal health coverage frameworks. The strategy also encourages countries to develop national policies that recognize traditional medicine while ensuring appropriate standards for patient safety and quality assurance (WHO, 2025).

At the continental level, the African Union and the African Medicines Agency (AMA) provide important opportunities for regulatory harmonization. The AMA is expected to support coordination of medicine regulation across African Union member states, including traditional and herbal medicinal products. Its role is particularly relevant in promoting consistent product registration standards, pharmacovigilance systems, regulatory capacity building, and cross-border cooperation in medicine governance. Regional economic communities also play an important role in harmonizing traditional medicine regulation. The Economic Community of West African States (ECOWAS), for example, has contributed to regional discussions on herbal medicine standards, pharmacopoeial development, and regulatory coordination. Such regional mechanisms are important because medicinal products, practitioners, and therapeutic knowledge often move across national borders, making isolated national regulation insufficient.

National legislation remains the foundation of ATM governance. Effective regulatory systems require clear legal provisions for practitioner registration, licensing, professional accountability, product registration, manufacturing standards, advertising control, pharmacovigilance, and protection of patients from unsafe or misleading practices. However, many countries still lack comprehensive legislation or struggle with implementation, enforcement, and coordination across health ministries, regulatory authorities, research institutions, and traditional practitioner associations (Mutombo et al., 2023; Ouma, 2022).

Country experiences illustrate this uneven policy landscape. Ghana has developed one of the more structured governance models, with formal policy frameworks, traditional medicine units in selected healthcare facilities, and institutions such as the Centre for Plant Medicine Research supporting herbal medicine development and institutional recognition. Nigeria has also established governance structures through the Nigerian Natural Medicine Development Agency, which supports traditional medicine research, product development, and policy coordination. In East Africa, Ethiopia formally recognizes traditional medicine within national health policy, while Kenya has introduced regulatory initiatives for herbal and complementary medicines through the Ministry of Health and the Pharmacy and Poisons Board (Ahlberg, 2017; Balcha, 2025; Gakuya et al., 2020; Vasconi and Owoahene-Acheampong, 2011).

Despite these developments, regulatory implementation remains uneven. Practitioner licensing and accreditation systems are inconsistent, product registration pathways are not uniformly applied, and traditional healers often continue to operate outside formal oversight. These gaps limit accountability and complicate coordination between traditional and biomedical sectors. Governance of ATM also intersects with intellectual property and protection of indigenous knowledge. Because much traditional medical knowledge is communal, orally transmitted, and culturally embedded, conventional intellectual property systems often provide inadequate protection. National and regional governance frameworks therefore need mechanisms for protecting indigenous knowledge, preventing biopiracy, and ensuring equitable benefit-sharing. Overall, policy and regulatory development for ATM requires harmonized legislation, stronger institutional coordination, practitioner licensing, product registration systems, pharmacovigilance mechanisms, and inclusion of indigenous knowledge holders in governance processes. Effective regulation should balance cultural recognition of traditional medicine with public accountability, patient protection, and coherent health system governance (Ikhoyameh et al., 2024; WHO, 2025).

## Ethical and legal considerations

The development, regulation, and commercialization of African Traditional Medicine (ATM) raise important ethical and legal questions concerning patient protection, indigenous knowledge systems, intellectual property rights, and equitable governance of biological and cultural resources. These issues are particularly significant because ATM exists at the intersection of healthcare, culture, biodiversity, and commercial innovation, where scientific, economic, and sociocultural interests frequently overlap. One major ethical consideration relates to informed consent and patient autonomy. Individuals utilizing traditional medicine should have access to clear and culturally appropriate information regarding the potential benefits, risks, limitations, and uncertainties associated with traditional therapies. Respect for patient autonomy requires that individuals are able to make informed healthcare decisions without coercion, misinformation, or stigmatization. This is especially important within pluralistic healthcare systems where patients may simultaneously use both traditional and biomedical treatments.

Concerns also arise regarding misrepresentation of efficacy and safety. Some traditional remedies may be promoted using unsupported therapeutic claims or without adequate scientific evaluation, particularly in the management of serious illnesses such as cancer, HIV/AIDS, diabetes, or mental health conditions. In certain situations, exclusive reliance on unvalidated therapies may contribute to delayed biomedical care, disease progression, or preventable complications. Ethical healthcare practice therefore requires balanced communication regarding both the potential value and limitations of traditional therapies while promoting patient safety and evidence-informed decision-making. Confidentiality and protection of patient information are additional ethical responsibilities within traditional healthcare practice. Although many traditional healing systems are community-based and relational in nature, maintaining patient privacy and professional confidentiality remains important for protecting trust and dignity within healthcare relationships. Strengthening ethical standards and professional accountability among both traditional and biomedical practitioners may therefore contribute to safer and more patient-centered healthcare systems.

Ethical considerations also extend to ownership and use of indigenous medical knowledge. In many African societies, medicinal knowledge is collectively held within families, clans, healer associations, or communities rather than by individual practitioners. Conventional intellectual property systems, which are largely based on individual ownership and written documentation, often inadequately recognize communal and orally transmitted knowledge systems. As a result, indigenous knowledge and biological resources have historically been utilized in pharmaceutical research and commercial development with limited recognition, participation, or compensation for local communities (Roche et al., 2017; Wong and Musa, 2025).

These concerns have contributed to ongoing debates regarding biopiracy, exploitation, and inequitable commercialization of medicinal resources. Ethical research and product development therefore require transparent collaboration with indigenous communities, culturally respectful engagement, and equitable governance frameworks capable of protecting traditional knowledge holders from exploitation. Benefit-sharing has consequently become an important principle within ethnopharmacological research and commercialization. Potential benefit-sharing mechanisms may include financial compensation, research collaboration, technology transfer, infrastructure development, capacity building, and community health investments. International frameworks such as the Nagoya Protocol on Access and Benefit-Sharing provide guidance regarding equitable use of genetic resources and associated traditional knowledge. However, implementation across many African countries remains uneven due to limitations in legal infrastructure, institutional capacity, documentation systems, and negotiation power among local communities (Bond and Scott, 2020; Sirakaya, 2022).

Broader legal and ethical challenges are also closely linked to historical and structural inequalities in global systems of knowledge production and pharmaceutical development. Colonial and postcolonial power dynamics have historically privileged Western scientific institutions while marginalizing indigenous systems of knowledge and healthcare practice. These inequities continue to influence whose knowledge is recognized, protected, commercialized, and institutionally supported within contemporary research and healthcare systems. Addressing ethical and legal challenges in ATM therefore requires more than regulatory oversight alone. It also requires inclusive governance models that recognize indigenous communities as legitimate knowledge holders and active participants in healthcare research, policy development, and innovation processes. Ethical integration of ATM into modern healthcare systems must balance scientific accountability, patient safety, cultural respect, intellectual property protection, and social justice considerations within increasingly pluralistic healthcare environments (WHO, 2025).

## Challenges

Despite increasing recognition of African Traditional Medicine (ATM) within healthcare policy and research, important scientific challenges continue to limit its broader integration into contemporary healthcare systems. Although many African medicinal plants demonstrate promising pharmacological activity, relatively few traditional therapies have undergone rigorous clinical evaluation, standardized formulation, or comprehensive toxicological assessment. Variability in plant composition, preparation methods, dosage, and quality control also complicates reproducibility and development of standardized therapeutic products.

Governance and institutional barriers remain equally significant. Regulatory systems for traditional medicine remain uneven across many African countries, with inconsistent practitioner licensing, fragmented legislation, limited pharmacovigilance infrastructure, and variable implementation of quality assurance standards. In addition, limited research funding, inadequate laboratory capacity, and weak interdisciplinary collaboration continue to constrain large-scale ethnopharmacological research and product development.

Epistemological and ethical challenges further complicate integration efforts. Indigenous healthcare systems continue to operate within historical knowledge hierarchies that privilege Western biomedical paradigms while marginalizing traditional therapeutic knowledge and cultural approaches to healing. At the same time, concerns related to intellectual property protection, biopiracy, benefit-sharing, patient safety, informed consent, and community ownership of indigenous knowledge remain insufficiently addressed within many policy and research frameworks.

Collectively, these challenges demonstrate that integration of ATM into modern healthcare systems requires coordinated scientific, regulatory, ethical, and institutional approaches capable of supporting safe, equitable, and culturally responsive healthcare systems.

## Future directions

Future development of African Traditional Medicine (ATM) should focus on building coordinated, evidence-informed, and culturally responsive healthcare systems capable of supporting safe integration of traditional and biomedical practices. Advancing ATM will require long-term collaboration among researchers, policymakers, healthcare institutions, regulatory agencies, and indigenous communities.

Strengthening interdisciplinary research should remain a major strategic priority. Future efforts should support multicenter clinical studies, ethnopharmacological research, toxicological evaluation, and development of standardized herbal formulations. Increased regional collaboration among African universities, research institutes, and public health institutions may improve scientific capacity and facilitate generation of context-specific evidence relevant to African healthcare systems.

Regulatory harmonization across African countries will also be important for improving governance of traditional medicinal products and practices. Institutions such as the African Medicines Agency (AMA), African Union, and regional economic communities may play important roles in supporting coordinated product registration systems, practitioner licensing frameworks, pharmacovigilance mechanisms, and harmonized quality assurance standards.

Future integration strategies should promote collaborative healthcare models that improve communication between traditional healers and biomedical practitioners while respecting cultural diversity and patient preferences. Structured referral systems, interdisciplinary training, and culturally informed healthcare policies may strengthen continuity of care within pluralistic healthcare environments.

Protection of indigenous knowledge systems should remain central to future policy and research agendas. Governance frameworks should promote equitable benefit-sharing, community participation, intellectual property protection, and ethical research partnerships that recognize indigenous communities as active stakeholders in healthcare innovation and medicinal knowledge preservation.

Sustainable management of medicinal plant biodiversity is equally important for the long-term future of ATM. Conservation strategies should integrate environmental protection, sustainable harvesting practices, community-based resource management, and preservation of indigenous ecological knowledge to safeguard medicinal resources for future generations.

Future scholarship on ATM should continue to adopt interdisciplinary approaches that integrate ethnopharmacology, medical anthropology, public health, pharmacology, ethics, and health systems research. Such approaches may contribute to more comprehensive understanding of traditional medicine and support development of balanced, scientifically rigorous, and culturally responsive healthcare systems across Africa.

## Conclusion

African Traditional Medicine (ATM) remains an important and enduring component of healthcare across the African continent, reflecting centuries of indigenous knowledge development, cultural practice, and community-based healing traditions. Rather than representing a single uniform system, ATM encompasses diverse therapeutic modalities, epistemological frameworks, and healthcare practices shaped by ecological, historical, cultural, and sociopolitical contexts. Its continued utilization within many African societies demonstrates both its social legitimacy and the persistence of pluralistic healthcare systems in which traditional and biomedical approaches frequently coexist.

This review highlights that the contemporary relevance of ATM extends beyond cultural heritage alone. Africa’s extensive medicinal biodiversity and longstanding ethnomedical knowledge provide important opportunities for ethnopharmacological research, natural-product discovery, and development of evidence-informed therapeutic interventions. Numerous African medicinal plants contain pharmacologically active compounds with antimicrobial, anti-inflammatory, antiparasitic, antioxidant, and immunomodulatory properties, supporting growing scientific interest in ATM as a potential contributor to healthcare innovation and pharmaceutical development.

At the same time, the review demonstrates that integration of ATM into formal healthcare systems remains complex and multidimensional. Historical processes of colonial disruption and epistemological marginalization continue to shape institutional recognition, research priorities, regulatory frameworks, and professional legitimacy of traditional medicine across Africa. Challenges related to scientific validation, standardization, quality control, pharmacovigilance, governance, intellectual property protection, and ethical oversight further complicate integration efforts.

Importantly, effective integration requires approaches that move beyond simplistic opposition between traditional and biomedical healthcare systems. ATM should not be evaluated solely through narrowly reductionist biomedical frameworks, nor should traditional knowledge systems be romanticized or exempted from scientific scrutiny and ethical accountability. Instead, balanced and interdisciplinary approaches are required that recognize both the therapeutic potential and the limitations of traditional medicine while respecting cultural context, patient autonomy, and evidence-informed healthcare practice.

The future development of ATM will therefore depend on strengthening ethnopharmacological research, clinical evaluation, regulatory harmonization, pharmacovigilance systems, and interdisciplinary collaboration across African healthcare institutions. Equally important is the protection of indigenous knowledge systems through equitable governance, community participation, biodiversity conservation, and fair benefit-sharing mechanisms capable of addressing historical and structural inequalities in global knowledge production and pharmaceutical development.

Ultimately, ATM occupies an important position within contemporary African healthcare landscapes as both a cultural institution and a potential contributor to healthcare innovation. Developing safe, equitable, scientifically rigorous, and culturally responsive models of integration will require sustained collaboration among researchers, policymakers, healthcare professionals, traditional practitioners, and indigenous communities. Such approaches may contribute not only to improved healthcare access and therapeutic development, but also to more inclusive and pluralistic health systems capable of responding to the diverse healthcare realities across Africa.

### Transparency statement

Language editing and structuring tools were used during manuscript preparation. All scientific content, interpretation, and conclusions were independently developed and critically reviewed by the author.

## References

[B1] Abdullahi A. A. (2011). Trends and challenges of traditional medicine in Africa. Afr. J. of Traditional, Complementary and Altern. Med. 8 (5S), 115–123. 10.4314/AJTCAM.V8I5S.5 22754064 PMC3252714

[B2] Agu P. C. Nduneseokwu N. C. Nwiziogo F. C. Okafor M. U. Alum E. U. Egwu C. O. (2025). Historical and ethnopharmacological perspectives on African medicinal plants: from traditional remedies to computational drug discovery. Sci. Afr. 30, e02941. 10.1016/J.SCIAF.2025.E02941

[B3] Ahlberg B. M. (2017). Integrated health care systems and Indigenous medicine: reflections from the sub-sahara African region. Front. in Sociol. 2, 12. 10.3389/fsoc.2017.00012

[B4] Akpuogwu M. O. Ngangah C. Donatus Nwoye C. (2025). Ethical theory and its practical implications in African traditional medicine. Int. J. of Res. and Sci. Innovation XII (VII), 1204–1212. 10.51244/ijrsi.2025.1215000100p

[B5] Ampomah I. G. Malau-Aduli B. S. Malau-Aduli A. E. O. Emeto T. I. (2020). Effectiveness of integrated health systems in Africa: a systematic review. Medicina 56 (6), 271. 10.3390/MEDICINA56060271 32486110 PMC7353894

[B6] Atanasov A. G. Zotchev S. B. Dirsch V. M. Orhan I. E. Banach M. Rollinger J. M. (2021). Natural products in drug discovery: advances and opportunities. Nat. Rev. Drug Discov. 2021 20 (3), 200–216. 10.1038/s41573-020-00114-z PMC7841765 33510482

[B7] Balcha A. (2025). Integration between Indigenous and conventional medicines in Ethiopia: unresolved historical conundrum. Integration between indigenous and conventional Medicines in Ethiop. J. of Afroasiat. Lang. 14 (1), 2025. 10.63469/jaal/1412

[B8] Berhe K. T. Gesesew H. A. Ward P. R. (2024). Traditional healing practices, factors influencing to access the practices and its complementary effect on mental health in Sub-Saharan Africa: a systematic review. BMJ Open 14 (9), e083004. 10.1136/BMJOPEN-2023-083004 39322598 PMC11429370

[B9] Bond M. R. Scott D. (2020). Digital biopiracy and the (dis)assembling of the Nagoya protocol. Geoforum 117, 24–32. 10.1016/j.geoforum.2020.09.001 33041359 PMC7536632

[B10] Degu A A. Terefe E. M. Some E. S. Tegegne G. T. (2022). Treatment outcomes and its associated factors among adult patients with selected solid malignancies at kenyatta national hospital: a hospital-based prospective cohort study. Cancer Manag. and Res. 14, 1525–1540. 10.2147/CMAR.S361485 35498512 PMC9042075

[B11] Degu S S. Meresa A. Animaw Z. Jegnie M. Asfaw A. Tegegn G. (2024). Vernonia amygdalina: a comprehensive review of the nutritional makeup, traditional medicinal use, and pharmacology of isolated phytochemicals and compounds. Front. in Nat. Prod. 3, 1347855. 10.3389/fntpr.2024.1347855

[B12] Devine S. N. O. Kolog E. A. Atinga R. (2022). Toward a knowledge-based system for African traditional herbal medicine: a design science research approach. Front. in Artif. Intell. 5, 856705. 10.3389/FRAI.2022.856705 PMC8959699 35355830

[B13] D’Almeida S. A. Gbomor S. E. Osaio-Kamara B. Olagunju M. T. Abodunrin O. R. Foláyan M. O. (2024). A scoping review of the use of traditional medicine for the management of ailments in West Africa. PLOS ONE 19 (7), e0306594. 10.1371/JOURNAL.PONE.0306594 38976677 PMC11230574

[B14] Etaware P. M. America oshua Egara O. W. Ekun V. S. (2025). Herbal medicine: scientific validation and future prospects. Int. J. of Pharm. and Chem. 11 (3), 67–75. 10.11648/J.IJPC.20251103.12

[B15] Feierman S. Janzen J. M. (1998). The social basis of health and healing in Africa

[B16] Gakuya D. W. Okumu M. O. Kiama S. G. Mbaria J. M. Gathumbi P. K. Mathiu P. M. (2020). Traditional medicine in Kenya: past and current status, challenges, and the way forward. Sci. Afr. 8, e00360. 10.1016/J.SCIAF.2020.E00360

[B17] Hooft A. Nabukalu D. Mwanga-Amumpaire J. Gardiner M. A. Sundararajan R. (2020). Factors motivating traditional healer versus biomedical facility use for treatment of pediatric febrile illness: results from a qualitative study in Southwestern Uganda. The Am. J. of Trop. Med. and Hyg. 103 (1), 501–507. 10.4269/AJTMH.19-0897 32458776 PMC7356444

[B18] Ikhoyameh M. Okete W. E. Ogboye R. M. Gbadebo O. S. Owoyemi O. K. (2024). Integrating traditional medicine into the African healthcare system post-Traditional medicine global summit: challenges and recommendations. PAMJ 47 (146), 146–147. 10.11604/PAMJ.2024.47.146.43011 38933435 PMC11204987

[B19] Kabumba B. (2023). Criminalizing Indigenous belief: the constitutional deficits of uganda’s witchcraft act. Oxf. J. of Law and Relig. 12 (1), 139–156. 10.1093/ojlr/rwad014

[B20] Karim M. R. Miletti-Gonzalez K. E. Aryee A. N. A. Besong S. A. (2025). Phytochemical analysis and antioxidant activities of Prunus africana bark, Leea indica and Paullinia pinnata leaf extracts. Antioxidants 14 (6), 666. 10.3390/antiox14060666 40563299 PMC12189430

[B21] Komakech R. Kang Y. Lee J. H. Omujal F. (2017). A review of the potential of phytochemicals from Prunus africana (Hook f.) kalkman stem bark for chemoprevention and chemotherapy of prostate cancer. Evidence-based Complementary and Altern. Med. 2017, 3014019. 10.1155/2017/3014019 28286531 PMC5327751

[B22] Krah E. de Kruijf J. Ragno L. (2018). Integrating traditional healers into the health care system: challenges and opportunities in rural northern Ghana. J. of Community Health 43 (1), 157–163. 10.1007/s10900-017-0398-4 28681282 PMC5767209

[B23] Lamwaka A. V. Obwona M. H. Lamwaka A. V. Obwona M. H. Onen D. Aber G. V. (2024). Barriers to global recognition of traditional African medicines: a systematic review of cultural, scientific, regulatory, and economic factors. Am. J. of Plant Sci. 15 (11), 1031–1048. 10.4236/AJPS.2024.1511066

[B24] Meddows-Taylor S. Ramadwa T. E. (2025). A comprehensive review of the traditional uses, pharmacological activity and phytochemistry of Warburgia salutaris in Southern Africa. South Afr. J. of Bot. 179, 134–146. 10.1016/j.sajb.2025.02.016

[B25] Mguni S. Mashinya F. Khabo-Mmekoa C. Shai L. J. (2023). A review of Zanthoxylum chalybeum Engl: ethnomedicinal uses, pharmacology, phytochemistry and toxicology. J. of Med. Plants for Econ. Dev. 7 (1), 10.4102/jomped.v7i1.202

[B26] Mthethwa W. Kalk E. Sylvester C. D. Ntatamala I. (2025). Decolonising undergraduate medical curricula through the integration of African traditional medicine and science - a South African perspective. Afr. J. of Health Prof. Educ. 17 (2), e1815. 10.7196/ajhpe.2025.v17i2.1815

[B27] Mutombo P. N. Kasilo O. M. J. James P. B. Wardle J. Kunle O. Katerere D. (2023). Experiences and challenges of African traditional medicine: lessons from COVID-19 pandemic. BMJ Glob. Health 8 (8), e010813. 10.1136/BMJGH-2022-010813 37558269 PMC10414097

[B28] Nagini S. Palrasu M. Bishayee A. (2024). Limonoids from neem (Azadirachta indica A. Juss.) are potential anticancer drug candidates. Med. Res. Rev. 44 (Issue 2), 457–496. 10.1002/med.21988 37589457

[B29] Nargawe L. Malathi G. C. S V. Upadhyay L. (2023). Ethnobotanical survey of medicinal plants used in traditional medicine in Africa. Plant Sci. Archives 8 (4), 26–28. 10.51470/PSA.2023.8.4.26

[B30] Noor A. I. O. Terefe E. M. (2025). Evaluation of the antidiarrheal activity of dichloromethane-methanol crude extract of the aerial parts of Croton kinondoensis (Euphorbiaceae) in Mice. PLOS One 20 (10), e0333527. 10.1371/JOURNAL.PONE.0333527 41042765 PMC12494273

[B31] Oluwafemi B. B. (2025). Bridging traditional and modern health systems: the role of digital health in traditional medicine integration in Nigeria. MedRxiv.

[B32] Ouma A. (2022). Intergenerational learning processes of traditional medicinal knowledge and socio-spatial transformation dynamics. Front. in Sociol. 7, 661992. 10.3389/FSOC.2022.661992/TEXT PMC9301457 35874449

[B33] Roche J. Trivedi R. Drani E. Bhattacharya A. Zeijden A. Serin T. (2017). “Traditional medicine sharing experiences from the field,” in ICHCAP and #HeritageAlive and Ichngoforum, Living Heritage Series UNESCO.

[B34] Saeed E. Javed F. Rana Z. Perveen R. Mallhi I. Y. Amjad I. (2025). Bioactive compounds, their mechanisms of action, and cardioprotective effects of pomegranate (Punica granatum): a comprehensive review. eFood 6 (Issue 4), e70075. 10.1002/efd2.70075

[B35] Sifuna N. Sifuna N. (2022). African traditional medicine: its potential, limitations and challenges. Afr. Traditional Med. Its Potential, Limitations and Challenges 5 (1), 141. 10.36959/569/475

[B36] Sirakaya A. (2022). Is the Nagoya protocol designed to conserve biodiversity? Plants People Planet 4 (1), 68–75. 10.1002/ppp3.10221

[B37] Terefe E. M. Okalebo F. A. Derese S. Langat M. K. Mas-claret E. Qureshi K. A. (2022). Anti-HIV ermiasolides from *Croton megalocarpus. Molecules* . 1–13. 10.3390/molecules27207040 PMC9610617 36296633

[B38] Vasconi E. Owoahene-Acheampong S. (2011). Recognition and integration of traditional medicine in Ghana A perspective. Res. Rev. of the Inst. of Afr. Stud. 26 (2). 10.4314/rrias.v26i2.63109

[B39] Wang M. Liu Z. Sun Y. Zhang Y. Ghaffar A. Ren M. (2025). Integration of traditional and complementary medicine into primary health care systems: a systematic review. Bull. of the World Health Organ. 103 (11), 675–684C. 10.2471/BLT.25.293465 41180277 PMC12578535

[B40] WHO (2025). Global Traditional Medicine Strategy 2025-2034. 10.2471/BLT.25.293414 PMC12578523 41180270

[B41] Wong B. K. M. Musa G. (2025). Health tourism in Asia: definition framework, issues, and way forward. Int. J. of Tour. Policy 15 (6), 475–489. 10.1504/IJTP.2025.150638

[B42] Yifru M. Tilahun A. Awoke A. Kassa Z. (2025). Traditional medicinal plant based remedies for common ailments in Menz Keya Gebreal District, North Shewa Zone, Ethiopia. BMC Complementary Med. and Ther. 25 (1), 411. 10.1186/S12906-025-05158-5 PMC12587719 41194202

